# Combined low-dose everolimus and low-dose tacrolimus after Alemtuzumab induction therapy: a randomized prospective trial in lung transplantation

**DOI:** 10.1186/s13063-020-04843-9

**Published:** 2021-01-04

**Authors:** Alberto Benazzo, Ara Cho, Anna Nechay, Stefan Schwarz, Florian Frommlet, Thomas Wekerle, Konrad Hoetzenecker, Peter Jaksch

**Affiliations:** Medizinische Universitat Wien, Vienna, Austria

**Keywords:** Lung transplantation, Alemtuzumab, Induction therapy, Everolimus, Kidney function, Immunotolerance

## Abstract

**Background:**

Long-term outcomes of lung transplantation are severely affected by comorbidities and development of chronic rejection. Among the comorbidities, kidney insufficiency is one of the most frequent and it is mainly caused by the cumulative effect of calcineurin inhibitors (CNIs). Currently, the most used immunosuppression protocols worldwide include induction therapy and a triple-drug maintenance immunosuppression, with one calcineurin inhibitor, one anti-proliferative drug, and steroids. Our center has pioneered the use of alemtuzumab as induction therapy, showing promising results in terms of short- and long-term outcomes. The use of alemtuzumab followed by a low-dose double drug maintenance immunosuppression, in fact, led to better kidney function along with excellent results in terms of acute rejection, chronic lung allograft dysfunction, and survival (Benazzo et al., PLoS One 14(1):e0210443, 2019). The hypothesis driving the proposed clinical trial is that de novo introduction of low-dose everolimus early after transplantation could further improve kidney function via a further reduction of tacrolimus. Based on evidences from kidney transplantation, moreover, alemtuzumab induction therapy followed by a low-dose everolimus and low-dose tacrolimus may have a permissive action on regulatory immune cells thus stimulating allograft acceptance.

**Methods:**

A randomized prospective clinical trial has been set up to answer the research hypothesis. One hundred ten patients will be randomized in two groups. Treatment group will receive the new maintenance immunosuppression protocol based on low-dose tacrolimus and low-dose everolimus and the control group will receive our standard immunosuppression protocol. Both groups will receive alemtuzumab induction therapy. The primary endpoint of the study is to analyze the effect of the new low-dose immunosuppression protocol on kidney function in terms of eGFR change. The study will have a duration of 24 months from the time of randomization. Immunomodulatory status of the patients will be assessed with flow cytometry and gene expression analysis.

**Discussion:**

For the first time in the field of lung transplantation, this trial proposes the combined use of significantly reduced tacrolimus and everolimus after alemtuzumab induction. The new protocol may have a twofold advantage: (1) further reduction of nephrotoxic tacrolimus and (2) permissive influence on regulatory cells development with further reduction of rejection episodes.

**Trial registration:**

EUDRACT Nr 2018-001680-24. Registered on 15 May 2018

## Administrative information

Note: the numbers in curly brackets in this protocol refer to SPIRIT checklist item numbers. The order of the items has been modified to group similar items (see http://www.equator-network.org/reporting-guidelines/spirit-2013-statement-defining-standard-protocol-items-for-clinical-trials/).

**Table:** 

Title {1}	**Combined low-dose everolimus and low-dose tacrolimus after alemtuzumab induction therapy: a randomized prospective trial in lung transplantation. Acronym: ALiKE**
Trial registration {2a and 2b}.	EudraCT number: **2018-001680-24**
Protocol version {3}	29.05.2020/version 4/ ethical review committee number 1458/2018.
Funding {4}	Peer-reviewed funding from *Austrian Science Fund*. Identifier: KLI 817-B.
Author details {5a}	1. Medical University of Vienna, Department of Surgery 2. Medical University of Vienna, Center for Medical Statistics, Informatics and Intelligent Systems
Name and contact information for the trial sponsor {5b}	Peter Jaksch, MD peter.jaksch@meduniwien.ac.at
Role of sponsor {5c}	The trial has no third-party sponsor. It is funded by the *Austrian Science Fund. The sponsor of the trial is the Medical University of Vienna and its representative is Peter Jaksch, who is also the PI of the trial. Alberto Benazzo is responsible for the design, collection, management,* analysis, and interpretation of data; he wrote the report and is responsible for the decision to submit the report for publication under the supervision of the PI Peter Jaksch

## Introduction

### Background and rationale {6a}

Lung transplantation is an established therapy for end-stage lung diseases; however, long-term outcomes are severely affected by comorbidities and by the development of chronic rejection. Kidney insufficiency is the comorbidity most frequently recorded after lung transplantation with an incidence of 50% at 5 years and 70% at 10 years. It is mainly caused by the cumulative nephrotoxic effect of calcineurin inhibitors (CNIs). Moreover, chronic rejection (chronic lung allograft dysfunction—CLAD) hampers long-term outcomes and poses a big financial burden. During the last years, significant efforts have been made to improve kidney function and reduce the occurrence of CLAD. With this purpose, different immunosuppression strategies have been developed. Currently, the most commonly used protocols include induction therapy and a triple-drug maintenance immunosuppression, with a CNI (mainly tacrolimus), an anti-proliferative drug (mainly MMF), and steroids.

#### Immunosuppression

##### Induction immunosuppression

The rationale underlying induction immunosuppression is twofold: (1) to reduce the dose of maintenance immunosuppression, thereby minimizing its adverse effects, and (2) to reduce the incidence of ACR, which is a known risk factor of CLAD. According to the 33rd report from the Registry of the ISHLT, approximately 50% of patients transplanted in the past 10 years received some type of induction therapy [1]. Currently, the majority of lung transplant centers use IL2R Antagonists (mainly basiliximab) with contrasting outcomes [2–6]. Basiliximab blocks the IL-2 binding site of CD25, inhibiting effector T cells and other proinflammatory cells. Polyclonal anti-lymphocyte preparations (ATG) are the second most used induction therapy. ATG-mediated mechanisms include apoptosis and anergy of T cells, antibody-dependent cellular cytotoxicity (ADCC), and complement-mediated lymphocyte lysis [7–9]. More recently, an increasing number of centers started using alemtuzumab, a humanized monoclonal antibody targeting CD52, as induction therapy. The receptor recognized by this antibody is expressed on several immune cells (T and B lymphocytes, natural killer (NK) cells and macrophages), and its activation induces cell lysis leading to immune cell depletion [10, 11]. B cell counts recover 3 to 6 months and T cell counts in 12 to 24 months after treatment [10, 12]. Few studies investigated the specific reconstitution of T and B cell compartment. In the T cell compartment, CD4^+^CD45RO^+^ effectory memory T cells preferentially reconstitute 12 to 24 months after depletion, constituting the predominant T cell subset [12–17]. According to Bouvy and colleagues, in fact, CD4^+^CD45RO^+^ T cells seem to be resistant to alemtuzumab-driven depletion and moreover, to some extent, CD4^+^CD45RA^+^ näive T cells may convert to a memory phenotype in a lymphopenic environment [15]. In parallel to the increase of the effector memory T cells, frequency of CD4^+^CD25^+^FoxP3^+^ Treg cells seems to increase after the first month after alemtuzumab depletion [15]. Clinical data are supportive for the expansion of Tregs in organ transplant recipients after treatment with alemtuzumab [18]. In kidney transplant recipients, Bloom et al. reported an increase of Tregs and of the ratio of Treg/Teff compared to the pre-transplant baseline [16]. This could not be found in patients after basiliximab induction [16]. Similarly, Noris and colleagues showed an increase of CD4^+^CD25^+^FoxP3^+^ Tregs in kidney transplant recipient treated with alemtuzumab and sirolimus up to 24 months after depletion therapy [12]. The same set of cells showed a hyporesponsive phenotype against alloantigens [12]. A similar reconstitution pattern was also found in the field of multiple sclerosis, where alemtuzumab is an established therapeutic agent for relapsing-remitting forms. In addition to a predominant CD4CD45^+^RO^+^ and CD4^+^CD25^+^FoxP3^+^ T cells, a clear increase of anti-inflammatory cytokines and a suppression of Th1 and Th17 transcription factors as well as pro-inflammatory cytokines was observed [13, 14]. In summary, it seems that alemtuzumab therapy is associated with higher levels of memory T cells and Tregs. Whether this phenomenon is due to a selective depletion or proliferation is still unclear. After reconstitution, the B cell compartment mainly consists of IgM-producing näive B cells and transient B cells [10, 12]. Such phenotypic changes in B cells play a crucial role in allograft tolerance mechanisms [19]. In stable immunosuppression-free kidney recipients, a higher percentage of this subset of B cells was found. Based on these findings it is tempting to speculate whether alemtuzumab induction therapy creates an immunological environment with regulatory characteristics. To date, published data on the use of alemtuzumab in lung transplantation are scarce. One of the biggest series was published in 2011 by Shyu and colleagues. In their 5-year experience, the alemtuzumab group showed improved patient and graft survival compared to the non-induction and daclizumab group. Moreover, freedom from ACR, lymphocytic bronchiolitis (LB), and bronchiolitis obliterans (BO) was improved [20]. Furuya and colleagues reviewed the UNOS database and identified 738 patients receiving alemtuzumab induction therapy [21]. These patients had a significant better survival and showed a lower risk for CLAD.

##### Maintenance immunosuppression

The current maintenance immunosuppressive protocols are calcineurin inhibitor-based “triple-drug” regimens [1], based on the combination of CNI, anti-proliferative agents and steroids.

CNIs are the main immunosuppressive agents used in lung transplantation. They are nephrotoxic, neurotoxic, and associated with other side effects [22, 23]. CNIs target the intracellular phosphatase calcineurin, which inhibits the nuclear translocation of the cytosolic nuclear factor of activated T cells (NFAT). NFAT activates IL-2 transcription, which regulates proliferation and maturation for all T cell subtypes, including Tregs [24]. The effects of CNIs on Tregs have been shown to be dose and duration dependent. High-dose but not low-dose CNI alters gene expression in Tregs [25]. Moreover, NFAT partly resides into the nucleus of Tregs, making them resistant to low-dose action of CNI [26–28]. Current clinical trials in the field of kidney transplantation evaluate the efficacy and safety of CNI-sparing protocols to reduce nephrotoxicity. The observation that Tregs are resistant to low-dose CNI and that the combination of CNI with mTOR inhibitors restores Tregs [25–28] suggest that whenever feasible the reduction of CNI could not only improve kidney function but also support Tregs development.

Sirolimus and everolimus are two newer anti-proliferative agents of the mTOR inhibitor class. Their role in lung transplantation, however, is still debated. Everolimus allows to efficiently maintain a stable systemic immunosuppression level in combination with lower tacrolimus dosage [29–33]. As a consequence, everolimus is usually introduced in the long-term follow-up to stop further deterioration of kidney function. The potential of this agent to prevent CLAD, however, is still matter of debate and contrasting results have been published. Nowadays, mTOR inhibitors are mainly used to reduce the dose of CNI, when kidney dysfunction starts to deteriorate. In addition, there is evidence showing that everolimus favors Tregs development over Tconvs [34–38]. This effect is dose-dependent and high doses of mTOR inhibitors may negatively influence Tregs function [39]. In kidney transplant recipients, for example, patients on sirolimus show a 4-fold increase in circulating Tregs when compared to patients receiving cyclosporine [40]. Moreover, a conversion from a CNI-based to a rapamycin-based regimen led to an increase of peripheral Tregs in kidney and liver recipients [41, 42]. This effect is dose-dependent and high doses of mTOR inhibitors may negatively influence Tregs function [39]. Crucial nodes for the development and homeostasis of Tregs are TCR and IL-2R engagement as well as the PI3K-Akt-mTOR signaling pathway [38]. Low-dose CNI may not harm Tregs by deprivation of IL-2 [26] while concomitantly may inhibit intranuclear translocation of cytoplasmatic NFAT in Teffs. Low-dose mTOR inhibition may mediate the transcription of Tregs-specific genes through the activation of Forkhead box O (Foxo) transcription factors and inhibition of mTORC1 and 2 [38]. Thus, a combined CNI and mTOR inhibition could drive reprogramming of gene expression towards a pro-tolerogenic signature.

## Objectives {7}

According to our preliminary experience, the use of alemtuzumab induction and the consequent reduction of CNI is associated with an improvement of glomerular filtration rate. However, long-term kidney function is still impaired by the steady cumulative kidney damage driven by tacrolimus. In parallel, despite excellent freedom from acute rejection, chronic rejection rate is still high with approximately 25% of patients at 5 years [43]. This trial proposes a new immunosuppression protocol based on the early administration of low-dose everolimus in the attempt of preventing CNI-driven kidney damage and stimulating allograft tolerance. The addition of de novo everolimus early after transplantation, in fact, has two potential advantages: (1) it allows a significant reduction of nephrotoxic tacrolimus and (2) together with alemtuzumab it could have a permissive influence on regulatory cells development with reduction of chronic rejection rate.

Our primary objective is to analyze the effect of the proposed low-dose immunosuppression protocol on kidney function. As secondary objectives we intend to assess the difference between the investigational and the control immunosuppression protocol in terms of freedom from ACR, AMR, and CLAD, overall survival, occurrence of de novo donor-specific antibodies (DSA), occurrence of CMV infections, and occurrence of other comorbidities (e.g., hypertension, dyslipidemia, diabetes, tremor). As experimental objectives, immunomodulatory cell subsets will be characterized overtime and gene expression will be profiled in both groups, to gain insight into the potential immunomodulatory mechanisms of the proposed low-dose immunosuppression.

## Trial design {8}

This trial is a non-blinded randomized prospective trial. Since the dose of immunosuppressive agents will be adapted to reach the blood trough levels, a blinding is not realistically possible. One hundred ten patients respecting the inclusion criteria and exclusion criteria will be randomized into two groups at postoperative day 30. In case of incomplete healing of bronchial anastomosis, patients will be reevaluated for inclusion between postoperative day 30 and 60. Each group will include 55 patients. Patients will be stratified according to glomerular filtration rate (GFR) values before randomization (≥ 40–60 ml/min/1.73m^2^, > 60–75 ml/min/1.73m^2^, or > 75–100 ml/min/1.73m^2^) and CMV status (low risk vs high risk). Such stratification will ensure to equally allocate patients at high risk of kidney dysfunction in the two groups.

## Methods: participants, interventions, and outcomes

### Study setting {9}

Medical University of Vienna, Department of Surgery, Division of Thoracic Surgery, Head: Walter Klepetko, MD

### Eligibility criteria {10}

Inclusion criteria:

1. Bilateral lung transplantation
2. Age > 18 years
3. Complete healing of bronchial anastomosis

Exclusion criteria:

1. Inclusion in other studies
2. Pre-transplant colonization with Burkholderia or resistant Mycobacterium abscessus
3. Wound infection at randomization

### Who will take informed consent? {26a}

Alberto Benazzo, MD

### Additional consent provisions for collection and use of participant data and biological specimens {26b}

Based on the guidelines of the ethical review committee, participants are asked to give consent for participation to trial, for collection and use of blood samples for scientific investigations and for dissemination of the results undersigning a single informed consent. The participants are not allowed to give limited consent.

## Interventions

### Explanation for the choice of comparators {6b}

The trial will compare a new maintenance immunosuppression protocol based on low-dose CNI and mTOR inhibitors with our standard of care, namely a maintenance immunosuppression based on CNI and steroid. The investigational medical products are tacrolimus (CNI) and everolimus (mTOR inhibitor).

### Intervention description {11a}

After transplantation, alemtuzumab will be given as a single dose of 30 mg on the intensive care unit (ICU). All included patients will receive maintenance immunosuppression protocol until randomization according to Table 1. After randomization, patients will be treated according to the protocol to the summarized in Table 2. According to current evidence, B cells reconstitute between 6 to 12 months after alemtuzumab induction. As a consequence, mycophenolate mofetil, an immunosuppressive agent also targeting B cells, will be introduced after 12 months to prevent incidence of acute humoral rejection.

**Table 1:** Immunosuppression protocol during the first month after LuTx is the same in the two groups

*Time after LuTx*	Tacrolimus trough blood level (ng/ml)	Aprednisolone (mg/kg)
1–2 months	8–10	0.2

**Table 2:** Immunosuppression protocol after randomization. In the treatment group, patients will receive a reduced dose of everolimus and tacrolimus whereas control patients will receive our standard immunosuppression. MMF will be introduced in both groups 12 months after transplantation

*Time after LuTx*	Tacrolimus trough blood level (ng/ml)		Everolimus trough blood level (ng/ml)		Aprednisolone (mg/kg)	MMF
	Control	Treatment	Control	Treatment	All patients	All patients
1–3 months	8–10	4–5	–	4–5	0.2	–
3–6 months	6–8	3–4	–	3–4	0.15	–
6–12 months	6–8	3–4	–	3–4	0.1	–
12–24 months	5–7	2.5	–	2.5	5 mg/d	1–1.5 g 2× a day

Perioperative infectious prophylaxis will be based on broad-spectrum antibiotics or adapted to resistance testing. All patients will receive a lifelong *Pneumocystis* prophylaxis with trimethoprim-sulfamethoxazole. Prophylactic inhalation therapy with amphotericin B and refobacin will be provided for 1–3 months. CMV prophylaxis will include CMV hyperimmunoglobulines (POD 1, 7, 14, and 21) together with valganciclovir for a minimum of 3 months. In high-risk patients (donor CMV IgG positive, recipient CMV IgG negative), a 12-month prophylaxis will be performed. Follow-up visits and pulmonary function test will take place once a month during the first year and later every 3 months. Bronchoscopy with transbronchial biopsy (TBB) and bronchoalveolar lavage (BAL) will be performed 1, 2, 3, 6, 12, 24 months after transplantation. A chest computed tomography (CT) will be done once a year. Biopsies will be classified according to ISHLT criteria [44]. ACR grade A2 and LB B2 or higher will be primarily treated with steroids for 3 days with consecutive dose tapering, and in case of non-response, ATG (2 mg/kg) will be administered for 5 days, followed by extracorporeal photopheresis (ECP) if necessary. Kidney insufficiency will be defined as GFR < 60 mL/min/1.73 m^2^ for ≥ 3 months. Infectious complications, including *Aspergillus* infection, will be defined as presence of pathogens with clinical signs and symptoms and need for specific treatment. Donor-specific antibodies (DSA) testing will be performed at each follow-up visit. AMR will be defined according to the ISHLT consensus. Patients with clinical AMR will be firstly treated by plasmapheresis followed by ECP.

### Criteria for discontinuing or modifying allocated interventions {11b}

The investigator must temporarily interrupt or permanently discontinue the study drug if continued administration of the study drug is believed to be contrary to the best interests of the patient. The interruption or premature discontinuation of study drug might be triggered by an adverse event (AE), a diagnostic or therapeutic procedure, an abnormal assessment (e.g., laboratory abnormalities), or for administrative reasons, in particular withdrawal of the patient’s consent.

If the reason for premature permanent discontinuation of study treatment is an AE, the patient should have a “premature end of study (EOS)” visit with all the assessments performed before the study drug discontinuation, whenever possible. If the patient experiences a life-threatening episode of acute rejection or infection requiring invasive mechanical ventilation or extracorporeal support, the patient will be withdrawn from the study and have the end of study (EOS) visit with all the assessments performed before the study drug discontinuation, whenever possible. If the patient develops a malignancy, the patient will be withdrawn from the study and have the end of study (EOS) visit with all the assessments performed before the study drug discontinuation, whenever possible.

Subjects may prematurely discontinue from the study at any time. Premature discontinuation from the study means that the subject did not undergo an end of study examination as planned per protocol.

Subjects must be withdrawn under the following circumstances:

1. At their own request
2. If the investigator feels it would not be in the best interest of the subject to continue
3. If the subject violates conditions laid out in the consent form/information sheet or disregards instructions by the study personal

The sponsor has the right to close this study at any time. The IEC and the competent regulatory authority must be informed within 15 days of early termination.

The trial will be terminated prematurely in the following cases:

1. If adverse events occur which are so serious that the risk-benefit ratio is no longer acceptable.
2. If the number of dropouts is so high that proper completion of the trial cannot realistically be expected.

### Strategies to improve adherence to interventions {11c}

During hospital stay, each IMP will be administered by nursing staff, so compliance issues are not relevant.

After discharge from the hospital, a patient diary will be used. In parallel, the patient will be asked to bring all the original drug packages at the follow-up visits, to check for the number of taken and remaining pills. The medical staff will record any incompliance on the database.

### Relevant concomitant care permitted or prohibited during the trial {11d}

Participation to other studies is not allowed.

Elective surgical procedures are allowed only after temporary discontinuation of mTOR inhibitors.

### Provisions for post-trial care {30}

During their participation in the clinical trial, the patients will be insured as defined by legal requirements. The investigator of the clinical trial will receive a copy of the insurance conditions of the “patients’ insurance.” The sponsor is providing insurance in order to indemnify (legal and financial coverage) the investigator/center against claims arising from the study, except for claims that arise from malpractice and/or negligence. The compensation of the subject in the event of study-related injuries will comply with the applicable regulations.

Patients’ insurance will be stipulated with Zürich Versicherungs AG as part of the contract with MedUniWien (Polizzennummer: 07229622-2)

Details on the existing patients’ insurance are given in the patient information sheet.

### Outcomes {12}

#### Primary endpoint

- eGFR calculated by the CKD-EPI formula at 24 months after transplantation

#### Secondary endpoints

- Incidence of acute cellular rejection
- Incidence of lymphocytic bronchiolitis
- Incidence of antibody-mediated rejection
- Incidence of chronic lung allograft dysfunction
- Incidence of dnDSA
- Survival

### Participant timeline {13}

**Table:** 

		Study period					
		Screening	Randomization	Follow-up			EOS visit
Timepoint**		***Evaluation for Tx***	***30 to 60 POD***	***3 months after Tx***	***6 months after Tx***	***12 months after Tx***	***24 months after Tx***
**Screening:**							
**Eligibility screen**		X					
**Informed consent**		X					
**Randomization:**			*X*				
**Immunosuppression:**							
**Control group:**	**TAC + steroids**			X	X	X	
	**TAC + MMF + steroids**						X
**Treatment group:**	**Low-dose TAC + EVR + steroids**			X	X	X	
	**Low-dose TAC + EVR + MMF + steroids**						X
**Clinical assessments:**							
**Blood examinations**				X	X	X	X
**24 h urinalysis**				X	X	X	X
**Pulmonary function tests**				X	X	X	X
**ECG**				X	X	X	X
**Chest CT scan**						X	X
**Bronchoscopy with TBB and BAL**				X	X	X	X
**HLA-screening**				X	X	X	X
**Histological analysis of TBB**				X	X	X	X
**Microbiological analysis of BAL**				X	X	X	X
**Blood CMV count**				X	X	X	X
**Experimental assessments:**							
**FACS of T and B cells and DC**		X				X	X
**Gene expression analysis**		X				X	X
**OUTCOMES:**							
**eGFR at EOS**							X
**Freedom from ACR**				X	X	X	X
**Freedom from CLAD**							X
**Overall survival**				X	X	X	X
**Occurrence of de novo DSA**				X	X	X	X
**Occurrence of CMV infections**				X	X	X	X
**Occurrence of other comorbidities**				X	X	X	X

### Sample size {14}

#### 110 participants, 55 per group

Sample size calculations are based on available retrospective data of 231 patients treated with our standard protocol (control group). eGFR data are shown for all these patients (left panel) and after 7 outliers were removed who had initial values of eGFR > 220 (right panel).

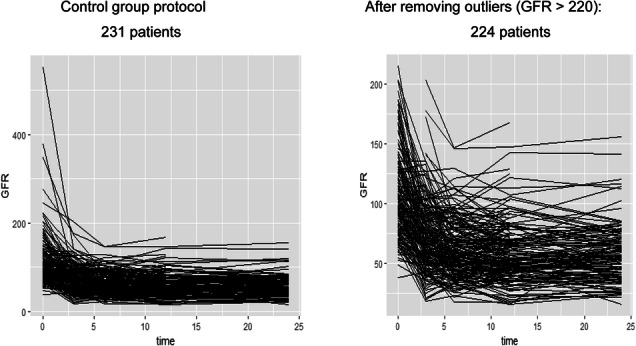



Our modeling approach assumes that for each patient eGFR strongly drops over the first 6 months and from then on only mildly decreases for the next 6 months. After 12 months, eGFR has stabilized and does not change for each patient till the end of the study after 24 months. The value obtained after 12 months for each patient is modeled as a random intercept. The decrease over the first 6 months differs significantly between patients and is therefore modeled by an additional random effect. To model the longitudinal data, we applied thus the following mixed effects model:

$$ {Y}_{ij}={\beta}_j+{\gamma}_{0i}+{\gamma}_{1i}{c}_j+{\epsilon}_{ij} $$

Index *i* refers to the *i*th individual and index *j* ∈ {1, …, 5} corresponds to the time points 0, 3, 6, 12, and 24 months, respectively. The fixed effects *β*_*j*_ model the average of eGFR at a given time point. ${\gamma}_{0i}\sim N\left(0,{\sigma}_o^2\right)$ is the random intercept and ${\gamma}_{1i}\sim N\left(0,{\sigma}_o^2\right)$ is the random slope for each individual. The (non-linear) random slope is defined by the vector *c* = [1, 0.5, 0.1, 0, 0] which allows each individual to have a different amount of decrease of eGFR within the first 6 months. Consequently, this model allows for more variance (and less correlation between time points) at the beginning of the trial. The final term ${\epsilon}_{ij}\sim N\left(0,{\sigma}_{\epsilon}^2\right)$ corresponds to the normally distributed error term. All random variables specified are assumed to be statistically independent.

Sample size calculations are based on an assumed effect size shown in the next graph. The blue line shows the estimated average behavior of GFR over time for the 224 patients after removing outliers. GFR stabilizes at 60 mL/min. The relevant treatment effect we want to detect is such that GFR stabilizes with the new immunosuppression protocol at 72 mL/min.

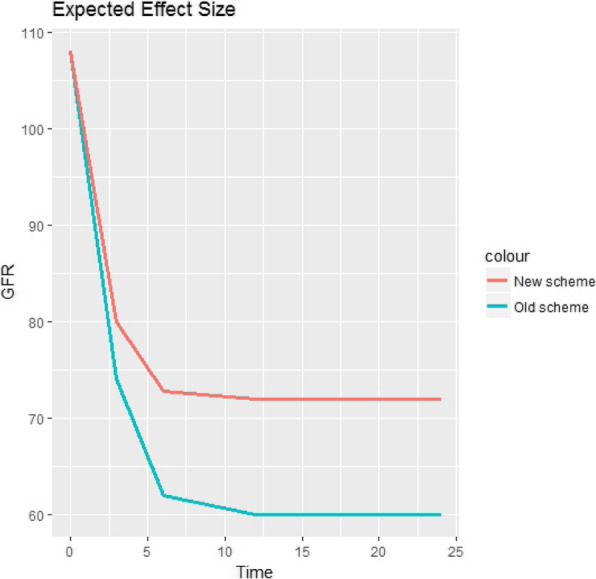



To make best use of the longitudinal data, we are not only testing the difference in GFR between the two treatment groups at 24 months or at 12 months (which could be accomplished by a simple ANCOVA taking the baseline value for each individual as a covariate) but we actually want to compare the whole curves for the two treatments. To this end, we specify a time-dependent effect vector of the form *C*_*eff*_ = [0, 0.5, 0.9, 1, 1] where our main endpoint is the interaction between *C*_*eff*_ and the treatment group. The idea is that initially there is no difference between the two groups while the effect is increasing over the first 6 months and has reached its full amount after 12 months. The following plot presents power curves for this contrast for different group sample sizes and compares with the power of simple ANCOVA analysis of GFR after 12 months or 24 months, respectively.

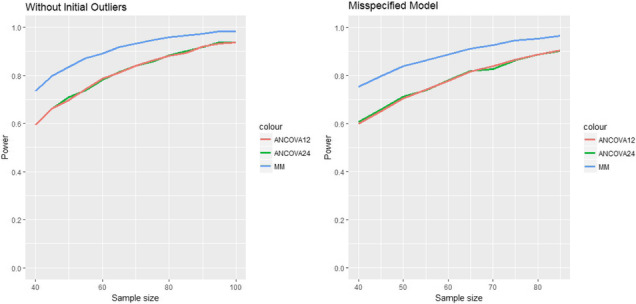



In the first plot, we simulated exactly under the model described above (the same random effects and the same contrast *C*_*eff*_). The second plot is based on simulations where both the vector *c* defining the random effect as well as the vector *C*_*eff*_ specifying the treatment effect were slightly changed. In both cases, the mixed model (1) was extended to test the interaction between *C*_*eff*_ and treatment at a significance level *α* = 0.05. The power curves (based on 5000 simulation replicates) indicate that the model is fairly robust against slight perturbations of these two vectors and that 45 patients per group are sufficient to detect the specified effect with a power of 80% (we assumed for both groups a covariance structure as given by the preliminary data for the control group). Considering a potential dropout rate of 20% results in a sample size of 55 per group.

### Recruitment {15}

All eligible patients who will be listed for lung transplantation will be asked to participate to the study. A selected team composed by transplant physicians, thoracic surgeons, and one psychologist will talk to the eligible patients at time of screening to explain the aim and design of the study. All possible benefits and risks will be explained to the eligible patients. Since the trial will run in a single-center, a personal relationship with the included patients will help to avoid any possible drop-out due to incompliance.

## Assignment of interventions: allocation

### Sequence generation {16a}

Randomization will be performed with an online software developed by the Medical University of Vienna and already successfully used in several clinical trials. Website: https://cemsiis.meduniwien.ac.at/en/kb/science-research/software/randomizer/

Patients will be stratified according to glomerular filtration rate (GFR) values before randomization (≥ 40–60 ml/min/1.73m^2^, > 60–75 ml/min/1.73m^2^, or > 75–100 ml/min/1.73m^2^) and CMV status (low risk vs high risk). Such stratification will ensure us to equally allocate patients at high risk of kidney dysfunction in the two groups.

### Concealment mechanism {16b}

Randomization will be sequentially numbered. Each patient will be assigned with an alphanumeric code.

### Implementation {16c}

Patients will be screened by Peter Jaksch and Alberto Benazzo. Peter Jaksch and Alberto Benazzo will check eligibility of the recipients at randomization time. Alberto Benazzo will perform randomization.

## Assignment of interventions: blinding

### Who will be blinded {17a}

Blinding is not realistically possible in this academic single-center trial. An open-label trial allows concentration-controlled administration of immunosuppressant agents, which have a narrow therapeutic index.

### Procedure for unblinding if needed {17b}

Blinding is not planned

## Data collection and management

### Plans for assessment and collection of outcomes {18a}

Data will be collected in an electronic case report form on CLINCASE platform (https://www.meduniwien.ac.at/web/mitarbeiterinnen/it-hilfe-support/it4science/plattformen/kss/). The platform has been developed by the Medical University of Vienna and is routinely used in clinical trial performed at our Institution. Data will be collected by Alberto Benazzo and a trained study nurse. All data will be directly taken from official medical reports. All reports will be printed and the correctness of data will be double-checked by Alberto Benazzo and Peter Jaksch. Moreover, an external monitoring will check the study course.

### Plans to promote participant retention and complete follow-up {18b}

Continuous personal contact with the patients will be established by Alberto Benazzo and our transplant psychologist in order to address possible compliance issue and drop-outs. Based on our preliminary data, a drop-out rate as high as 20% could be expected.

Patients who interrupt the study will undergo the following examinations:

- Blood examinations (differential blood count, electrolytes and chemistry, kidney and liver function tests, CRP, LDL, HDL, triglycerides, cholesterol and coagulation status)
- 24 h urinalysis
- Pulmonary function tests
- ECG
- Chest CT scan
- Bronchoscopy with TBB and BAL
- HLA-screening
- Histological analysis of TBB
- Microbiological analysis of BAL
- Blood CMV count

### Data management {19}

A subject screening and identification Log will be completed for all enrolled subjects with the reasons for exclusion. For this trial, an electronic CRF will be used. Only the investigator or designated sub-investigators will have access to the CRF. A CRF will be created for each subject enrolled. This also applies to those subjects who fail to complete the study. If a subject withdraws from the study, the reason must be noted on the CRF. Case report forms are to be completed on an ongoing basis. In CRF, demographics of the patients, inclusion criteria, randomization group, the medical information from each follow-up visit, adverse events or reason for withdrawal from the study and the EOS visit will be reported. CRF entries and corrections will only be performed by study site staff authorized by the investigator. Data collected at all visits are entered into an interactive form. The eCRFs will be verified source documents, following guidelines established before study onset as detailed in the monitoring plan. The investigator will maintain adequate and accurate records to enable the conduct of the study to be fully documented and the study data to be subsequently verified (according to ICH-GCP “essential documents”). These documents will be classified into two different categories: investigator’s study site file (ISF) with all essential documents regarding the study conduct, and subjects’ clinical source documents. Subjects’ clinical source documents include all patient hospital clinical records in original version. These two categories of documents must be kept on file by the investigator for as long as needed to comply with the regulatory requirements. The designated monitor will contact and visit the investigator on a regularly basis and will be allowed to have direct access to all source documents needed to verify the entries in the CRFs and other protocol-related documents, provided that confidentiality is maintained in agreement with local regulations. It will be the monitor’s responsibility to inspect the CRFs at regular intervals according to the monitoring plan throughout the study, to verify the adherence to the protocol and the completeness, consistency, and accuracy of the data being entered on them. Monitoring visits will be performed every 3 months. Monitoring will be performed by Clinical Trials Coordination Centre. Upon request, the investigator will make all study-related source data and records available to a qualified quality assurance auditor mandated by the sponsor or to competent authority inspectors. The main purposes of an audit or inspection are to confirm that the rights and welfare of the subjects have been adequately protected and that all data relevant for assessment of safety and efficacy of the investigational product have appropriately been reported to the sponsor.

### Confidentiality {27}

All patients’ data will be collected and stored with an alphanumeric code. The code key will be kept in safe custody by the PI and by the personnel designated by the PI. Ethical review committee, national and European health authorities can have access to the data. The investigator will maintain adequate and accurate records to enable the conduct of the study to be fully documented and the study data to be subsequently verified (according to ICH-GCP “essential documents”). These documents must be kept on file by the investigator for as long as needed to comply with the regulatory requirements.

### Plans for collection, laboratory evaluation, and storage of biological specimens for genetic or molecular analysis in this trial/future use {33}

Collected blood will be immediately processed and will be used for FACS analysis and gene expression analysis. Additional blood and BAL sample will be collected and stored as plasma, serum, DNA, and BAL sample in our institutional biobank.

## Statistical methods

### Statistical methods for primary and secondary outcomes {20a}

#### Primary endpoint analysis

An *F*-test for the primary endpoint using mixed model analysis will be performed using the function lme from the R package nlme.

#### Secondary endpoint analysis

Simple ANCOVA analysis for GFR after 12 months and after 24 months, respectively, will be performed.

Chi-square tests, Fisher exact tests, Mann-Whitney *U* tests, or ANOVA will be used to compare differences between the groups. Curves for survival will be generated with Kaplan-Meier method and compared by log-rank tests. A univariate and multivariable Cox regression will be performed to find predictors of survival and CLAD incidence.

### Interim analyses {21b}

No interim analysis is planned.

### Methods for additional analyses (e.g., subgroup analyses) {20b}

No additional analyses are planned.

### Methods in analysis to handle protocol non-adherence and any statistical methods to handle missing data {20c}

#### Two different analysis sets are defined

The (modified) intention-to-treat set includes all randomized patients for which measurements are available at least at time points 0 and 3 months.

The per-protocol set comprises all randomized patients for which measurements are available at least at time points 0 and 3 months and who did not violate the protocol in a way that might affect the evaluation of the effect of the immune suppression regimen on the primary objective, i.e., without major protocol violations.

Since the presented trial is a monocentric study and the follow-up will entirely occur in our center, we do not expect any kind of missing data.

### Plans to give access to the full protocol, participant-level data, and statistical code {31c}

Access to the data is restricted to the PI and designated personnel. Ethical review committee, national and European health authorities have access to the data. Additional access will be allowed only after written permission by the Medical University of Vienna. The data will be visible only after pseudonymization.

## Oversight and monitoring

### Composition of the coordinating center and trial steering committee {5d}

Composition of the coordinating center:

Peter Jaksch: screening and randomization of the patients, follow-up, responsible for data correctness.

Alberto Benazzo: screening and randomization of the patients, follow-up, responsible for data correctness.

Thomas Wekerle: supervision of experimental investigations

Ara Cho: responsible for FACS analysis and RNA extraction

Anna Nechay: screening of the patients, responsible for data collection in eCRF

Monika Meixner: responsible for data collection, correctness of the data, communication with competent authorities

Florian Frommlet: responsible for statistical design and statistical analysis

Endpoint adjudication committee

AB and PJ will adjudicate endpoints. Monitoring committee will proof correctness of endpoint adjudication.

Data management team

Peter Jaksch

Alberto Benazzo

Anna Nechay

Monika Meixner

### Composition of the data monitoring committee, its role and reporting structure {21a}

Monitoring will be performed by Clinical Trials Coordination Centre (https://kks.meduniwien.ac.at/). Data monitoring committee (DMC) will be composed by two project managers and will be supervised by the deputy head of the Clinical Trials Coordination Centre. The designated monitor will contact and visit the investigator on a regularly basis and will be allowed to have direct access to all source documents needed to verify the entries in the CRFs and other protocol-related documents provided that subject confidentiality is maintained in agreement with local regulations. It will be the monitor’s responsibility to inspect the CRFs at regular intervals according to the monitoring plan throughout the study, to verify the adherence to the protocol and the completeness, consistency, and accuracy of the data being entered on them. Monitoring visits will be performed every 3 months.

### Adverse event reporting and harms {22}

Adverse events will be continuously collected and assessed throughout the clinical trial at follow-up visits. Moreover, patients included in the patients may contact the study staff through a 24/7 active phone line. Each adverse event will be reported in the eCRF.

The following details will be entered:

- Type of adverse event
- Start (date and time)
- End (date and time)
- Severity (mild, moderate, severe)
- Serious (no/yes)
- Unexpected (no/yes)
- Outcome (resolved, resolving, not resolved, resolved with sequelae, unknown, fatal)
- Relation to study drug (related/probably/possibly/unlikely/not related/not assessable),

In case of a serious adverse, a written report is also to be prepared and should at least contain the following:

- Patient number
- Patient: sex
- The suspected investigational medical product (IMP)
- The adverse event assessed as serious
- Short description of the event and outcome

If applicable, the initial report should be followed by the follow-up report, indicating the outcome of the SAE.

The regulatory authorities and the Institutional Review Board/Independent Ethics Committee (IRB/IEC) must be informed about all suspected unexpected serious adverse reactions (SUSAR). Such reports shall be made by the sponsor and should content at least the following details:

- Patient number (study code/screening number)
- Patient: age in years, sex
- Name of investigator and investigating site
- Period of administration
- The suspected investigational medical product (IMP)
- The adverse event assessed as serious and unexpected and for which there is a suspected causal relationship to the IMP
- Concomitant disease and medication
- Short description of the event:
- Description
- Onset and, if applicable, end
- Therapeutic intervention
- Causal relationship
- Seriousness criteria or reportable reason

Electronic reporting should be the expected method for reporting of SUSARs to the competent authority. In that case, the format and content as defined by the regulatory requirements should be adhered to. The latest version of MedDRA should be applied. Lower level terms (LLT) should be used.

In case an adverse event occurs, the investigator has to use all supportive measures for best patient treatment. Management of adverse event will differ according to severity and causality. Admission or transfer to our hospital will be reevaluated on a daily basis. Moreover, treatment study drugs will be transiently interrupted in case of a clear causality could be confirmed.

### Frequency and plans for auditing trial conduct {23}

Monitoring will be performed by Clinical Trials Coordination Centre.

DMC will contact and visit the investigator on a regularly basis and will be allowed to have direct access to all source documents needed to verify the entries in the CRFs and other protocol-related documents, provided that subject confidentiality is maintained in agreement with local regulations. It will be the monitor’s responsibility to inspect the CRFs at regular intervals according to the monitoring plan throughout the study, to verify the adherence to the protocol and the completeness, consistency, and accuracy of the data being entered on them. Monitoring visits will be performed every 3 months.

Monitoring visits will include verification of following data:

1. Source data,
2. SAE/safety reports,
3. Informed consent,
4. Drug accountability,
5. Query management,
6. Investigator site file,
7. Other essential documents.

Upon request, the investigator will make all study-related source data and records available to a qualified quality assurance auditor mandated by the sponsor or to competent authority inspectors. The main purposes of an audit or inspection are to confirm that the rights and welfare of the subjects have been adequately protected and that all data relevant for assessment of safety and efficacy of the investigational product have appropriately been reported to the sponsor.

### Plans for communicating important protocol amendments to relevant parties (e.g., trial participants, ethical committees) {25}

#### Protocol amendments

Proposed amendments will be submitted to the appropriate competent authorities (CA) and ethic commission (EC). Substantial amendments may be implemented only after CA/EC approval has been obtained. Amendments that are intended to eliminate an apparent immediate hazard to subjects may be implemented prior to receiving CA/EC approval. However, in this case, approval must be obtained as soon as possible after implementation.

## Dissemination plans {31a}

In order to disseminate results, we plan the following activities: (a) edition of at least 3 scientific papers; (b) presentation of obtained data to international meeting of different scientific societies (e.g., ISHLT, ERS, ATS, ESOT); (c) organization of a dissemination meeting with patient-organizations on lung transplantation in Austria, in particular with a no-profit partner-organization called “Lunge Aktiv,” which deals with awareness on end-stage lung diseases and quality of life after transplantation.

## Discussion

The Vienna Lung Transplant Program has pioneered the use of alemtuzumab as induction therapy in lung transplantation together with the groups from Pittsburgh and St. Louis, showing promising results in terms of short- and long-term outcomes. Our center, moreover, conducted the only open-label randomized prospective trial published to date comparing alemtuzumab and ATG. In this study, a significant reduction of higher-grade rejection rates was shown in the alemtuzumab group [45]. Kidney insufficiency and CLAD are responsible for poor long-term survival and for the reduced quality of life for lung recipients. Since, to date, no established immunosuppression strategy is available to prevent deterioration of kidney function, the reduction of nephrotoxic CNI and implementation of nephroprotective mTOR inhibitors may represent an effective immunosuppressive strategy. Moreover, we expect that the combination of alemtuzumab with low-dose tacrolimus and low-dose everolimus has the additional effect of inducing a pro-tolerogenic immunomodulatory environment in the recipients.

Our project will address unmet medical needs and innovative aspects for the following reasons: (1) due to the high immunogenicity of lung tissue, the required immunosuppression level after lung transplantation is one of the highest among all solid organ transplantation. This is associated with high incidence of CNI-related side effects. Our protocol could provide a meaningful reduction of the cumulative immunosuppression dose leading to a significant decrease of long-term comorbidities deriving from CNI therapy. (2) According to experimental evidence and clinical experience, low-dose everolimus as well as alemtuzumab induction are associated with a tolerogenic immune cell phenotype. Moreover, it was speculated that CNIs, inhibiting IL-2 production, may reduce Tregs proliferation. Based on this knowledge, our clinical protocol may have the potential to improve allograft acceptance and reduce risk for CLAD. (3) T regulatory cells are heightened in lung transplant recipients with stable graft function and counteract type 17 immune response against self-antigens [46–49]. However, to date, no systematic study has investigated the main immunomodulatory cell subsets involved in lung transplantation. For the first time, our study will be able to provide a comprehensive evaluation of immunomodulatory cells over time and regulation of gene expression of lung recipients both on the waitlist and during follow-up. Most importantly, it can provide mechanistic insight into the immunomodulatory effects of CNIs, mTOR inhibitors, and alemtuzumab and their association with graft acceptance.

## Trial status

Registry: EudraCT

Database number: 2018-001680-24

URL of trial registry record: https://www.clinicaltrialsregister.eu/ctr-search/search?query=2018-001680-24

Trial status: recruiting

Protocol version date: 29.05.2020

Protocol version number: 4

Date recruitment start: 12 October 2018

Planned completion of recruitment: 01 October 2021

## Data Availability

Data belong to the Medical University of Vienna, and only the investigators have access to the final trial dataset. Since the clinical trial is an academic trial without involvement or support of third profit parties, no contractual restrictions limit access for the investigators to the data.

## Abbreviations

AATS: American Association of Thoracic Surgery
ACR: Acute cellular rejection
ADCC: Antibody-dependent cellular cytotoxicity
ADR: Adverse drug reaction
AE: Adverse event
ATG: Anti-thymocyte globulin
BAL: Bronchoalveolar lavage
BO: Bronchiolitis obliterans
Bregs: Regulatory B cells
CD: Cluster of differentiation
CLAD: Chronic lung allograft dysfunction
CMV: Cytomegalovirus
CNI: Calcineurin inhibitors
Coll(V): Collagen V
CRF: Case report form
CRO: Clinical research organization
CRP: C-reactive protein
CSR: Clinical study report
CT: Computer tomography
CYP: Cytochrome
DC: Dendritic cells
DOH: Declaration of Helsinki
DSA: Donor-specific antibodies
DSUR: Development safety update report
ECG: Electrocardiography
ECMO: Extracorporeal membrane oxygenation
eGFR: Estimated glomerular filtration rate
ELISA: Enzyme-linked immunosorbent assay
EOS: End of study
EP: Endpoint
ERS: European Respiratory Society
ESOT: European Society of Organ Transplantation
EU: European Union
FACS: Fluorescence-activated cell sorting
FMO: Fluorescence minus one
FoxP3: Forkhead box P3
GCP: Good Clinical Practice
HDL: High density lipoprotein
HLA: Human leukocyte antigen
ICU: Intensive care unit
IEC: Independent Ethics Committee
IFN-γ: Interferon-γ
IgG: Immunoglobulin G
IgM: Immunoglobulin M
IL-10: Interleukin-10
IL-17: Interleukin-17
IL2R: Inteleukin-2 receptor
IL-4: Interleukin 4
IMP: Investigational Medicinal Product
ISF: Investigator site file
ISHLT: International Society of Heart and Lung Transplantation
KKS: Koordinationszentrum für Klinische Studien
LB: Lymphocytic bronchiolitis
LDL: Low density lipoprotein
LuTx: Lung transplantation
mAb: Monoclonal antibody
MHC: Major histocompatibility complex
MMF: Mycophenolate mofetil
mTOR: Mechanistic target of rapamycin
NFAT: Nuclear factor of activated T cells
NK: Natural killer
PBMC: Peripheral blood mononuclear cell
PD-L1: Programmed death-ligand 1
Pi3k: Phosphoinositid-3-kinases
PML: Progressive multifocal leukoencephalopathy
POD: Postoperative day
PRES: Posterior reversible encephalopathy syndrome
PVAN: Polyoma virus-associated nephropathy
SAE: Serious adverse event
SAP: Statistical analysis plan
SAR: Serious adverse reaction
SOP: Standard operating procedure
SUSAR: Suspected unexpected serious adverse reaction
T2-MZP: Transitional 2 marginal zone precursor
TBB: Transbronchial biopsy
Tconv: Conventional T cells
TGF-β: Transforming growth factor-β
Th: T helper cells
TMF: Trial master file
Tregs: Regulatory T cells
UNOS: United network for organ sharing
